# KPC-3-Producing *Klebsiella pneumoniae* in Portugal Linked to Previously Circulating Non-CG258 Lineages and Uncommon Genetic Platforms (Tn*4401d*-IncFIA and Tn*4401d*-IncN)

**DOI:** 10.3389/fmicb.2016.01000

**Published:** 2016-06-28

**Authors:** Carla Rodrigues, Jan Bavlovič, Elisabete Machado, José Amorim, Luísa Peixe, Ângela Novais

**Affiliations:** ^1^UCIBIO/REQUIMTE, Laboratório de Microbiologia, Faculdade de Farmácia, Universidade do PortoPorto, Portugal; ^2^Faculty of Pharmacy in Hradec Králové, Charles UniversityPrague, Czech Republic; ^3^Faculty of Military Health Sciences, University of DefenseBrno, Czech Republic; ^4^FP-ENAS/CEBIMED, Faculdade de Ciências da Saúde, Universidade Fernando PessoaPorto, Portugal; ^5^Botelho Moniz Análises ClínicasSanto Tirso, Portugal

**Keywords:** multidrug resistance, carbapenemases, international clones, ST15, ST147, cointegrated plasmids, ColE

## Abstract

KPC-3-producing bacteria are endemic in many countries but only recently became apparent their wide distribution in different Portuguese hospitals. The aim of this study is to characterize genetic backgrounds associated with *bla*_KPC−3_ among *Klebsiella pneumoniae* isolates recently identified on non-hospitalized patients in Portugal. Twenty KPC-producing *K. pneumoniae* identified between October 2014 and November 2015 in three different community laboratories were characterized. Isolates were mainly from patients from long-term care facilities (*n* = 11) or nursing homes (*n* = 6), most of them (75%) previously hospitalized in different Portuguese hospitals. Standard methods were used for bacterial identification and antibiotic susceptibility testing. Carbapenemase production was assessed by the Blue-Carba test, and identification of *bla* genes was performed by PCR and sequencing. Epidemiological features of KPC-producing *K. pneumoniae* included population structure (*Xba*I-PFGE, MLST and *wzi* sequencing), genetic context (mapping of Tn*4401*), and plasmid (replicon typing, S1-PFGE, and hybridization) analysis. All *K. pneumoniae* isolates produced KPC-3, with two MDR *K. pneumoniae* epidemic clones representing 75% of the isolates, namely ST147 (*wzi64*/K14.64, February–November 2015) and ST15 (two lineages exhibiting capsular types *wzi19*/K19 or *wzi93*/K60, July-November 2015). Other sporadic clones were detected: ST231 (*n* = 3; *wzi*104), ST348 (*n* = 1; *wzi94*) and ST109 (*n* = 1, *wzi22*/K22.37). *bla*_KPC−3_ was identified within Tn*4401d* in all isolates, located in most cases (80%) on cointegrated plasmids (*repA*_FIA_+*repA*_FII_+*ori*_ColE1_;105-250 kb) or in 50 kb IncN plasmids. In conclusion, this study highlights a polyclonal structure of KPC-3-producing *K. pneumoniae* and the predominance of the ST147 clone among non-hospitalized patients in Portugal, linked to platforms still unnoticed in Europe (*bla*_KPC−3_-Tn*4401d*-IncFIA) or firstly reported (*bla*_KPC−3_-Tn*4401d*-IncN). This scenario underlines the recent penetration of successful mobile genetic elements in previously circulating MDR *K. pneumoniae* lineages (mainly ST147 and ST15) in Portugal, rather than the importation of the global lineages from clonal group 258.

## Introduction

In the last years, carbapenem-resistant *Enterobacteriaceae* have spread globally, being responsible for high rates of morbidity and mortality among healthcare-associated infections, mainly due to the depletion of effective therapeutic options (WHO, 2014; Albiger et al., 2015, http://www.cdc.gov/drugresistance/threat-report-2013/). After the first strain identified in 1996 in a North Carolina hospital (USA; Yigit et al., 2001), *Klebsiella pneumoniae* carbapenemases (KPCs) have exploded worldwide predominantly among *K. pneumoniae* isolates (Munoz-Price et al., 2013; Chen et al., 2014b). To date, 23 KPC variants (KPC-2 to KPC-24) have been described (http://www.lahey.org/Studies/other.asp#table1), being KPC-2 and KPC-3 the most widespread variants with variable geographic distribution (Munoz-Price et al., 2013; Nordmann and Poirel, 2014). While in some countries (USA, Colombia, Italy, and Israel) both KPC-2- and KPC-3-producing bacteria are endemic, in others (Argentina, Brazil, Greece, Poland, and China) KPC-2 producers are predominant (Munoz-Price et al., 2013; Albiger et al., 2015).

The *bla*_KPC_ genes are commonly located on Tn*4401*, a 10 kb Tn*3*-like transposon delimited by two 39-bp imperfect inverted repeat sequences harboring *bla*_KPC_, transposase and resolvase genes, and insertion sequences IS*Kpn7* (upstream *bla*_KPC_) and IS*Kpn6* (downstream *bla*_KPC_; Chen et al., 2014b). It is recognized as a highly active transposon enhancing the spread of *bla*_KPC_ genes to different plasmid scaffolds (Cuzon et al., 2011). To date, six Tn*4401* isoforms have been described with variable deletions between IS*Kpn7* and *bla*_KPC_ providing different promoter regions to the gene (*a*, −99 bp; *b*, no deletion; *c*, −215 bp; *d*, −68 bp; *e*, −255 bp; *g*, equal to isoform *c* but with one single nucleotide mutation on P2 promotor), and consequently different expression levels of the *bla*_KPC_ gene (Naas et al., 2012; Chmelnitsky et al., 2014). Besides its genetic environment, other factors are known to have greatly contributed to the spread of KPC producers in many countries, leading to an increasing challenge in the design of effective infection control measures. First, the introduction and subsequent expansion of *bla*_KPC−2_ and *bla*_KPC−3_ on multidrug resistant (MDR) *K. pneumoniae* lineages from clonal group (CG) 258 [sequence types (ST) 11, 258, 512] (Munoz-Price et al., 2013; Chen et al., 2014b), followed in a few countries (e.g., Israel, Italy, Colombia) by subsequent dispersion to other clonal backgrounds (Baraniak et al., 2015; Bonura et al., 2015; Ocampo et al., 2015). Second, the acquisition of *bla*_KPC_ by plasmids from different incompatibility groups (IncFII_*K*2_, IncFIA, IncI2, IncN, IncX3, ColE), favored a quick intra- and inter-species dissemination (Chen et al., 2014b).

In Portugal, KPC-2 was identified only in an environmental *Escherichia coli* isolate in 2010 (Poirel et al., 2012), while KPC-3 producers were first detected in 2009 in a central hospital (Machado et al., 2010). However, only recently became evident the widespread distribution of KPC-3 among *K. pneumoniae* isolates in different Portuguese hospitals (Silva et al., unpublished data; Manageiro et al., 2015). In this study, we aim to trace the landscape of KPC-3-producing *K. pneumoniae* isolates recently identified outside hospital boundaries in Portugal by detailed characterization of clonal and plasmid genetic backgrounds.

## Materials and methods

### Bacterial isolates and epidemiological data

Thirty *K. pneumoniae* isolates showing reduced susceptibility to carbapenems were identified between October 2014 and November 2015 in three different community laboratories in the North of Portugal, one of them receiving samples from all over the country. Twenty of them were identified as KPC producers and further characterized in this study. They were detected in urine samples (*n* = 19) or sputum (*n* = 1) of patients between 61 and 89 years old (mean age = 83; 16 females, 4 males; Table 1). Most of these patients were institutionalized in long-term care facilities (LTCFs) [*n* = 11; 55%; six different LTCFs (A–F)] or nursing homes (NH) [*n* = 6; 30%; five different NH (A–E)], while some were identified in ambulatory (*n* = 3; 15%; Table 1). Most of them (*n* = 13; 65%) had been hospitalized in the previous month in different hospitals from the North or Centre of Portugal, although in three cases no hospitalization or older hospitalization events (4–9 months) were detected (Table 1). Travel history abroad was discarded for 60% of the patients (*n* = 12/20), or considered improbable for the remaining patients due to their clinical conditions (impaired mobilization and chronic underlying diseases).

**Table 1:** **Epidemiological data of ***K. pneumoniae*** isolates carrying ***bla***_**KPC−3**_-Tn***4401d*** identified in non-hospitalized patients in Portugal (October 2014–November 2015)**.

**ST (no.)**	**PFGE-type (no.)**	***wzi*/K-type^a^**	**Date of isolation (month/year)**	**Source (no.)^b^**	**Local of previous hospitalizations (no)^c^**	**Age (range)**	**Gender**	**Sample (no.)**	**Plasmids associated with *bla*KPC−3 (size; Inc groups) (no.)**	**Other β-lactamases^d^**
ST147 (10)	Kp1 (1)	*wzi*64/K14.64	Feb–Nov/2015	LTCF A (2)	Hospital B (1), unknown (1)	88–89	F	Urine	~130 kb; FIA+FII+ColE (6)	(OXA-9), SHV-11 or SHV-28, (TEM-1)
				LTCF B (1)	unknown	89	F	Urine		
				LTCF C (1)	Hospital D	76	F	Urine		
				LTCF E (1)	Different hospitals	86	F	Urine		
				LTCF F (1)	Hospital E	81	M	Urine		
				NH A (2)	Hospital A	78–82	F, M	Urine	~50 kb; N (4)	(OXA-9), SHV-11
				NH D (1)	Hospital C^e^	85	F	Urine		
				Ambulatory (1)	Hospital A	77	F	Urine		
ST15 (5)	Kp2 (4)	*wzi*19/K19	Jul–Nov/2015	LTCF A (3)	Hospital B (2)^f^	83–84	F	Urine (2), sputum (1)	~130–140 kb; FIA+FII+ColE (4)	OXA-9, SHV-28, (TEM-1)
				LTCF D (1)	Hospital B	88	F	Urine		
	Kp3 (1)	*wzi*93/K60	Sep/2015	Ambulatory (1)	Hospital F	72	M	Urine	~135 kb; FIA+FII+ColE (1)	CTX-M-15, OXA-9, SHV-28
ST231 (3)	Kp4 (1)	*wzi*104/K-	Oct/2015	NH B (1)	Hospital A	88	F	Urine	~250 kb; FIA+FII+ColE (1)	SHV-1, (TEM-1)
				NH E (1)	Hospital G	89	F	Urine		
				Ambulatory (1)	Hospital C^e^	83	F	Urine		
ST348 (1)	Kp5 (1)	*wzi*94/K-	Oct/2014	NH C (1)	unknown	87	M	Urine	~105 kb; FIA+FII+ColE (1)	CTX-M-15, SHV-11, OXA-1, TEM-1
ST109 (1)	Kp6 (1)	*wzi*22/K22.37	Sep/2015	LTCF B (1)	unknown	61	F	Urine	~130 kb; FIA+FII+ColE (1)	SHV*new* (N196S)^g^

a *K-type is reported according with wzi allele-serotype associations reported by Brisse et al. (2013)*.

b *LTCFs A–D and NH A–C are located in the North region of Portugal, while LTCFs E–F and NH D-E are located in the Centre region of the country*.

c *Hospitals A, B, and D are located in the North region of Portugal, while Hospitals C, E, F, and G are located in the Centre region of the country*.

d *Variability among isolates is shown in parenthesis*.

e *Older hospitalization events (4–9 months)*.

f *No previous hospitalizations in one of the patients*.

g *GenBank accession number KX421191*.

*LTCF, long-term care facility; NH, nursing home; F, female; M, male*.

### Antibiotic resistance phenotypes and genotypes

Bacterial identification and preliminary antibiotic susceptibility testing were performed by Vitek II system (BioMérieux, Marcy l'Étoile, France). Confirmatory and additional tests for β-lactams (amoxicillin-clavulanic acid, mecillinam, cefoxitin, extended-spectrum cephalosporins, aztreonam, carbapenems), aminoglycosides (amikacin, gentamicin, netilmicin, tobramycin), fluoroquinolones (ciprofloxacin), folate pathway inhibitors (trimethoprim, trimethoprim-sulfamethoxazole), chloramphenicol, fosfomycin, and colistin were assessed by standard disc diffusion (Oxoid Ltd., Basingstoke, United Kingdom), agar dilution (for fosfomycin; in the presence of glucose-6-phosphate at 25 mg/L), broth microdilution (for colistin) or E-test (for carbapenems) (Liofilchem, Italy) methods according to EUCAST (www.eucast.org).

Production of carbapenemases was assessed by the Blue-Carba test (Pires et al., 2013), and identification of carbapenemases (*bla*_NDM_, *bla*_VIM_, *bla*_IMP_, *bla*_KPC,_*bla*_OXA−48_),or other *bla* genes (*bla*_CTX−M_, *bla*_SHV_, *bla*_TEM_, *bla*_OXA_) was performed by PCR and sequencing (Curiao et al., 2010; Bogaerts et al., 2013; Rodrigues et al., 2014).

### Population structure analysis

Population structure characterization included *Xba*I-Pulsed-Field Gel Electrophoresis (PFGE) (electrophoresis conditions: 5–20 s for 4 h and 25–50 s for 18 h, 14°C, 6 V/cm^2^), and multi-locus sequence typing (MLST) (http://bigsdb.web.pasteur.fr/klebsiella/primers_used.html) in representative isolates, as described (Rodrigues et al., 2014). Molecular capsule typing was performed by PCR and sequencing of *wzi* gene (Brisse et al., 2013).

### Characterization of the genetic environment and location of *bla*_KPC_ genes

The genetic context of *bla*_KPC−3_ was investigated by PCR and sequencing targeting Tn*4401* conserved sequences (Chen et al., 2014a). Location of *bla* (*bla*_KPC−3_, *bla*_CTX−M−15_) genes and plasmid characterization were assessed by S1- and I-*Ceu*I-PFGE and identification of replication genes by PCR, sequencing and hybridization (Carattoli et al., 2005; García-Fernández et al., 2009; Villa et al., 2010; Chen et al., 2014a; Rodrigues et al., 2014).

## Results

### Carbapanemase production and variable antibiotic resistance phenotypes

All isolates produced KPC-3 and demonstrated resistance or intermediate phenotypes to ertapenem (MIC = 1 to 16 mg/L), and susceptible, intermediate or resistance phenotypes to imipenem (MIC = 2 to 16 mg/L) and meropenem (MIC = 1 to 8 mg/L), with colonies growing within the inhibition zone of all carbapenems tested, a hetero-resistance phenotype usually observed for KPC producers (Nordmann et al., 2009; Table 2). Although for some isolates the MIC values for imipenem and meropenem were interpreted as susceptible by the clinical breakpoints defined by EUCAST, in all cases they were above the epidemiological cut-off values (ECOFFs) defined for *K. pneumoniae* (http://www.eucast.org/mic_distributions_and_ecoffs/; Table 2).

**Table 2:** **Antimicrobial resistance patterns of KPC-3-producing ***K. pneumoniae*** clones**.

**Antimicrobial**		**% of Resistance (MIC range, mg/L)**^a^, ^b^					
	**All**	**ST147**	**ST15-Kp2**	**ST15-Kp3**	**ST231**	**ST348**	**ST109**
	**(*n* = 20)**	**(*n* = 10)**	**(*n* = 4)**	**(*n* = 1)**	**(*n* = 3)**	**(*n* = 1)**	**(*n* = 1)**
Amoxicillin/clavulanic acid	100	100	100	100	100	100	100
Mecillinam	100	100	100	100	100	100	100
Ceftazidime	100	100	100	100	100	100	100
Cefotaxime	100	100	100	100	100	100	100
Cefepime	100	100	100	100	100	100	100
Cefotaxime	85	80	100	100	100	100	0
Aztreonam	100	100	100	100	100	100	100
Ertapenem	100 (1–16)	100 (1–4)	100 (4–16)	100 (8)	100 (4–8)	100 (8)	100 (1)
Imipenem	75 (2–16)	100 (4–8)	25 (2–8)	100 (8)	33 (2–8)	100 (16)	100 (4)
Meropenem	40 (1–8)	20 (1–4)	25 (2–4)	100 (8)	67 (2–8)	100 (4)	100 (4)
Amikacin	50	60	75	0	33	0	0
Gentamicin	70	70	100	0	67	100	0
Netilmicin	75	70	100	100	67	100	0
Tobramycin	80	70	100	100	100	100	0
Ciprofloxacin	95	100	100	100	100	100	0
Sulfamethoxazole/trimethoprim	90	80	100	100	100	100	100
Trimethoprim	90	80	100	100	100	100	100
Chloramphenicol	35	0	75	100	100	0	0
Fosfomycin	15	10	0	100	0	100	0
Colistin	0	0	0	0	0	0	0

a *All intermediate isolates were considered as resistant*.

b *Clinical Breakpoints (Ertapenem - S ≤ 0.5 mg/L; Imipenem and Meropenem - S ≤ 2 mg/L) and ECOFF values (Ertapenem - WT ≤ 0.064 mg/L; Imipenem - WT ≤ 1 mg/L; Meropenem - WT ≤ 0.125 mg/L) for MIC defined by EUCAST for K. pneumoniae*.

All isolates were defined as multidrug resistant (MDR) in accordance with the definition of MDR for *Enterobacteriaceae* (non-susceptible to ≥1 agent in ≥3 antimicrobial categories; Magiorakos et al., 2012), although some of them exhibited a less extensive resistance profile to non-β-lactams, being resistant only to ciprofloxacin (Table 2). All isolates were susceptible to colistin (MIC = 0.25–2 mg/L; Table 2).

### KPC-3 was identified among locally circulating *K. pneumoniae* clones

KPC-3-producing *K. pneumoniae* isolates were assigned to six different PFGE-types (arbitrarily designated as Kp1 to Kp6), each one of them linked to a specific capsular type (Table 1). Most isolates belonged to ST147 carrying *wzi*64 (K14.64; *n* = 10 Kp1, 50%; detected between February to November 2015) and produced additionally SHV-11 (*n* = 9) or SHV-28 (*n* = 1), OXA-9 (*n* = 6) and/or TEM-1 (*n* = 1; Table 1). Some (*n* = 4) of these patients had recently been hospitalized in hospitals (A and D) where KPC-3-producing ST147 isolates exhibiting the same PFGE-type were detected (data not shown, Silva et al., unpublished data). ST15 was also frequent (*n* = 5, 25%), with two different clones being identified (*n* = 4 Kp2 carrying *wzi*19/K19; and *n* = 1 Kp3 carrying *wzi*93/K60) between July and November 2015. These isolates co-produced OXA-9 and SHV-28. All ST15-Kp2 were identified in patients from the same LTCF or region, and in one case there was no previous hospitalization (Table 2). ST231 isolates carrying *wzi*104 (*n* = 3 Kp4; October 2015) co-produced SHV-1 and TEM-1 (*n* = 1), and were identified in patients for which no epidemiological link could be established. Sporadic clones such as ST348 carrying *wzi*94 (*n* = 1 Kp5; October 2014) or ST109 carrying *wzi*22 (K22.37; *n* = 1 Kp6; September 2015) producing other β-lactamases were also detected (Table 1).

### IncFIA and IncN plasmids involved in the dissemination of *bla*_KPC−3_-*Tn4401d*

In all isolates, the *bla*_KPC−3_ was identified between IS*Kpn7* (upstream) and IS*Kpn6* (downstream), in a structure previously described as Tn*4401* isoform *d*, known to have a 68bp deletion between IS*Kpn7* and *bla*_KPC_ gene (Chen et al., 2014b). Isolates showed a variable number of plasmids (2–5 plasmids) with different sizes (40–500 kb), frequently from IncFII_K_ and IncR families. In most of the cases (*n* = 16/20 isolates from different clones), *bla*_KPC−3_ was located within cointegrated plasmids (105 to 250 kb) carrying *repA*_FIA_(100% identity with that of pBK30661 plasmid, GenBank accession number KF954759), *repA*_FII_ (100% identity with that of pBK30683 plasmid, GenBank accession number KF954760), and *ori*_ColE1_ (100% identity with *ori* p15 gene pKBuS13 plasmid, GenBank accession number KM076933). In the remaining isolates (*n* = 4/20 isolates belonging to ST147), *bla*_KPC−3_ was identified in a ca. 50 kb IncN plasmid [*rep*_N_ showing 100% identity with that of pKPC_FCF/3SP plasmid (defined by pMLST as *repN* allele 7; ST15), GenBank accession number CP004367] (Table 1). The two isolates for which a less extended resistance profile was observed (Table 2) carried *bla*_KPC−3_ within IncN plasmids and no additional IncF plasmids were observed. The *bla*_CTX−M−15_ (when present) was variably located in a ca. 200 kb-IncFII_K7_ (ST348) or in a ca. 60 kb-IncR (ST15-Kp3) plasmid type.

## Discussion

In this study, we highlight a polyclonal structure of KPC-3 producing *K. pneumoniae* isolates among patients outside hospital boundaries in Portugal consistent with nosocomial acquisition, and unveil novel or uncovered plasmid backbones carrying *bla*_KPC−3_ in Europe.

The first clinical cases of KPC-3 producers in Portugal were detected in 2009 in a pediatric unit of a hospital from the Lisbon and Tagus Valley region and involved 2 *K. pneumoniae* belonging to ST11 (Machado et al., 2010). Months later and until 2011, an outbreak involving 41 KPC-3-producing isolates, most of them (*n* = 29) assigned to ST14, was reported (Calisto et al., 2012). More recently, a nationwide study reported 22 *K. pneumoniae* producing KPC-3 (mainly ST11, ST14, ST15, and ST147 clones) in several hospitals between 2010 and 2013 but plasmid backgrounds had been poorly characterized (Manageiro et al., 2015). However, the situation concerning carbapenemase-producing *Enterobacteriaceae* in Portugal was only recently recognized in the EuSCAPE survey, where our country appeared in level 2b (sporadic hospital outbreaks) mainly due to the expansion of KPC producers (Albiger et al., 2015). The data presented in this study strengthens an ongoing dissemination of KPC-3 producers in Portugal, where the identification of these bacteria among such a high diversity of healthcare institutions other than hospitals might potentiate their impact for both hospital and community settings. It is thus advisable a reinforcement of infection control measures, surveillance, and tracking of isolates resistant to carbapenems in clinical institutions, and a coordinated action between clinicians, epidemiologists and national reference laboratories for guidance and harmonization of protocols (Albiger et al., 2015).

We observed a polyclonal structure of KPC-3-producing *K. pneumoniae* isolates, where most of the clones identified (ST15, ST147, ST348) exhibited the same PFGE-pattern as CTX-M-15 (ST348, ST15-Kp2, and -Kp3) or SHV-12 (ST147) producers previously involved in hospital- and community-acquired infections at least in the North region of Portugal (2010–2012; Rodrigues et al., unpublished data; Rodrigues et al., 2014). These “high-risk clones” have been linked to the worldwide expansion of different ESBL (CTX-M-15 and different SHV-types) and carbapenemases (KPC, VIM, NDM, OXA-48-like), including in Portugal (Rodrigues et al., 2014). This scenario suggests recent acquisition of *bla*_KPC−3_ by MDR *K. pneumoniae* genetic lineages that were already circulating in Portugal (ST15, ST147, ST231, ST348), a situation observed less frequently than the amplification of CG258 lineages (Baraniak et al., 2015; Bonura et al., 2015; Ocampo et al., 2015).

The ST147 clone [clonal group (CG) 147] exhibiting capsular type K14.K64 was identified in patients from diverse LTCFs and NHs for a long period of time and seems to be the predominant lineage among KPC-3-producing *K. pneumoniae* in different healthcare settings (Silva et al., unpublished data; Manageiro et al., 2015). In fact, identical KPC-3-producing ST147 isolates were recently involved in outbreaks in hospitals where some of the patients had been previously hospitalized. Although, nosocomial acquisition is the most probable origin for most KPC-3 producers identified in patients included in this study, it is of notice that in three cases no obvious hospitalization link could be established. Indeed, considering the frequent displacement of these patients between institutions (integrated network of LTCFs in Portugal) and hospitals (we had only access to the last hospitalization event) and that intestinal colonization might be persistent in time (Feldman et al., 2013), we cannot completely discard cross transmission events in the units analyzed.

The identification of two distinct ST15 (CG15) lineages in this study (ST15-K19 and ST15-K60) is in line with recent studies based on *wzi*-capsule typing unveiling the circulation of distinct lineages within this CG, that might have differences in their relative occurrence, geographical, or niche distribution and/or host susceptibility (Bialek-Davenet et al., 2014; Holt et al., 2015; Rodrigues et al., 2015; Zhou et al., 2015, http://bigsdb.web.pasteur.fr/klebsiella/klebsiella.html). The ST231 *K. pneumoniae* clone (CG231) had already been linked to GES-5 plus SHV-12 production in Portugal (Manageiro et al., 2015) and its association with community invasive infections (sepsis, lethal pneumonia, or meningitis), and high content in virulence and antimicrobial resistance genes have recently been highlighted (Holt et al., 2015). The ST109 clone is rarely reported (http://bigsdb.web.pasteur.fr/klebsiella/klebsiella.html) and it is described for the first time in Portugal. However, it belongs to the CG17, associated with the expansion of CTX-M-15 and different carbapenemases worldwide (Rodrigues et al., 2014; Holt et al., 2015).

The *bla*_KPC−3_ was linked to Tn*4401d* isoform in all characterized KPC-3-producing *Enterobacteriaceae* from Portugal (this study; Manageiro et al., 2015). In this study, we show that in most cases (80%) Tn*4401d*-*bla*_KPC−3_ was located within cointegrated FIA, FII, and ColE1 plasmids (105–250 kb; Table 1) corroborating the strong association between Tn*4401d*-*bla*_KPC−3_ with IncFIA plasmids pointed out previously in large collections from the USA (Chen et al., 2014a; Deleo et al., 2014; Bowers et al., 2015; Chavda et al., 2015). We detected FIA and FII replicons identical to those of pBK30683 plasmid (GenBank accession number KF954760) plus an additional *ori*_ColE1_ gene identical to that of the ColE1 plasmid pKBuS13 (GenBank accession number KM076933), supporting the role of these mobilizable plasmids in the assembly of MDR plasmids (Chen et al., 2014a; Garbari et al., 2015). These and other (IncFIA plus IncA/C_2_ or IncFIA plus IncX_3_) cointegration forms seem to play an important role in the intra- and inter-species spread of carbapenem resistance genes (Chen et al., 2014a,b; Chavda et al., 2015). To the best of our knowledge, we unveil for the first time a cointegrate IncFIA platform carrying Tn*4401d*-*bla*_KPC−3_ in Europe, characterized and highly represented by far only in isolates from the USA (mainly among non-ST258 and non-*K. pneumoniae* isolates; Chen et al., 2014a). In the remaining isolates (20%, 4 ST147), *bla*_KPC−3_-Tn*4401d* was located on ca. 50 kb IncN plasmids, an association primarily described in this study.

In conclusion, this study highlights a polyclonal structure among KPC-3 producers identified in geographically dispersed non-hospitalized patients in Portugal, not always linked to nosocomial acquisition, a situation that deserves close monitoring due to its high clinical or epidemiological impact. In all cases, a common platform (*bla*_KPC−3_-Tn*4401d*) was identified in plasmids still unnoticed in Europe (*bla*_KPC−3_-Tn*4401d*-IncFIA) or firstly reported here (*bla*_KPC−3_-Tn*4401d*-IncN). Their identification in previously circulating MDR *K. pneumoniae* lineages in our area (ST147, ST15, ST231, ST348) underlines the recent penetration of successful mobile genetic elements in locally circulating clonal backgrounds, rather than the importation of the most common global lineages from CG258.

## Author contributions

CR and JB performed the experiments and contributed with the acquisition of molecular data. CR and ÂN wrote the article and performed analysis and interpretation of molecular data. EM and JA contributed with epidemiological data and revision of the manuscript. ÂN and LP contributed with the design of the study and final revision of the manuscript. All authors read and approved the final version of the manuscript.

## Funding

This work received financial support from the European Union (FEDER funds) through Programa Operacional Factores de Competitividade – COMPETE and National Funds (FCT, Fundação para a Ciência e Tecnologia) (UID/Multi/04378/2013). ÂN and CR were supported by fellowships (grant number SFRH/BPD/104927/2014 and SFRH/BD/84341/2012, respectively) from FCT through Programa Operacional Capital Humano (POCH).

### Conflict of interest statement

The authors declare that the research was conducted in the absence of any commercial or financial relationships that could be construed as a potential conflict of interest.
